# Analyzing the Impact of COVID-19 Lockdowns on Violent Crime

**DOI:** 10.3390/ijerph192315525

**Published:** 2022-11-23

**Authors:** Lin Liu, Jiayu Chang, Dongping Long, Heng Liu

**Affiliations:** 1 Center of Geoinformatics for Public Security, School of Geography and Remote Sensing, Guangzhou University, Guangzhou 510006, China; 2 Department of Geography, University of Cincinnati, Cincinnati, OH 45221-0131, USA

**Keywords:** COVID-19, pandemic risk rating, COVID-19 outbreak prevention and control policy, crime change, violent crime

## Abstract

Existing research suggests that COVID-19 lockdowns tend to contribute to a decrease in overall urban crime rates. Most studies have compared pre-lockdown and post-lockdown periods to lockdown periods in Western cities. Few have touched on the fine variations during lockdowns. Equally rare are intracity studies conducted in China. This study tested the relationship between violent crime and COVID-19 lockdown policies in ZG City in southern China. The distance from the isolation location to the nearest violent crime site, called “the nearest crime distance”, is a key variable in this study. Kernel density mapping and the Wilcoxon signed-rank test are used to compare the pre-lockdown and post-lockdown periods to the lockdown period. Panel logistic regression is used to test the fine variations among different stages during the lockdown. The result found an overall decline in violent crime during the lockdown and a bounce-back post-lockdown. Violent crime moved away from the isolation location during the lockdown. This outward spread continued for the first two months after the lifting of the lockdown, suggesting a lasting effect of the lockdown policy. During the lockdown, weekly changes in COVID-19 risk ratings at the district level in ZG City also affected changes in the nearest crime distance. In particular, an increase in the risk rating increased that distance, and a drop in the risk rating decreased that distance. These findings add new results to the literature and could have policy implications for joint crime and pandemic prevention and control.

## 1. Introduction

During the early stages of the COVID-19 pandemic, many countries adopted various lockdown measures, such as reductions in travel and assembly, closures of businesses, isolating infected people and sites, and quarantining exposed people. In China, these measures have been effective in curbing the spread of COVID-19, but they have also affected people’s daily lives [1,2,3]. The municipal government of ZG, China, also adopted lockdown policies to contain the spread of COVID-19. In 2020, the residential buildings of infected people and the surrounding neighborhoods were put into isolation, and the overall COVID-19 risk ratings of the entire district were elevated. When there were no confirmed cases or no new confirmed cases for 14 consecutive days, the district was considered “low risk”. If there were newly confirmed cases within 14 days and the cumulative number of confirmed cases did not exceed 50, or if the cumulative number of confirmed cases exceeded 50 but no aggregated outbreak occurred within 14 days, the district was considered “medium risk”. When the cumulative number of cases exceeded 50 and an aggregated outbreak occurred within 14 days, the district was considered “high risk”. An elevation to medium risk would automatically lead to the implementation of lockdown policies, including the closure of all entertainment establishments in the area, such as drinking bars and karaoke televisions bars (KTVs); the canceling of indoor gatherings; and the stopping of in-person classes. The policies of other Chinese cities are virtually the same as those adopted by ZG City.

Many studies have examined crime in the context of the COVID-19 pandemic [2,4,5,6,7,8,9,10,11,12,13,14]. Campedelli et al. [6] assessed the impact of quarantine policies on crime using a Bayesian time series and found that crimes, such as robbery, burglary, assault, and battery, decreased significantly under quarantine policies. Miyar et al. [8] found that most types of crime followed a U-shaped pattern of change, with an initial drop and a later return to a pre-pandemic level when the lockdown was lifted in Mexican municipalities. The study by Andresen and Hodgkinson [7] also showed that crime rates decreased significantly in the early days of the lockdown, but they began to rise when social restrictions were relaxed. Similarly, Payne et al. [12] used a seasonal autoregressive integrated moving average (SARIMA) model to predict expected crime trends in Queensland, Australia, in 2020 in the absence of a pandemic lockdown and found that most expected crimes were higher than the actual crime rate. Yang et al. [14] used a seasonal-trend decomposition procedure to identify crime trends during the COVID-19 period over time and found that there was a definite phased pattern of increasing trends in Chicago for the four crime categories of burglary, assault, battery, and robbery, with crime decreasing and then increasing in 2020. All of the aforementioned studies found an overall decrease in crime rates in the early parts of the COVID-19 pandemic, followed by a bounce-back.

There are also inconsistencies in the literature. Nivette et al. [2] discussed the changes in average crime rates in 27 cities worldwide through a meta-regression analysis and found that the trends were not consistent across crime types. Ashby [4] has shown that aggravated assault in public spaces did not change significantly, but the impact of the COVID-19 pandemic on crime varied across 16 cities. Calderon and Kaufman [5] found an increase in homicides and suicides under the impact of quarantine measures in 2020. Meanwhile, Mohler et al. [9] found an increase in violent crime in Los Angeles and Indianapolis following the COVID-19 pandemic. However, Payne et al. [12] spotted a decrease in the total number of aggravated assaults, nondomestic violent crimes, and sexual assaults in Australia. Moise and Piquero [10] identified clusters and outliers of violent crime using local spatial correlation statistical indicators and temporal alignment statistics and found clusters of violent crime in Miami-Dade County, Florida, in 2018 and 2019, but not in 2020. Scherg [13] further found a large decline in violent crime in areas with nighttime bars during the COVID-19 pandemic. In addition, Boman and Gallupe [15] argued that the reduction in violent crime under the impact of the COVID-19 pandemic was found in less-serious, group-type violent crimes, such as trespassing and vandalism, but rates for more serious crimes, such as homicide and domestic violence, remained unchanged or increased.

Existing studies have analyzed the mechanisms by which the built and social environments act on individual offenders, finding that the accessibility of areas and the spatial perceptions of offenders, neighborhood affluence, and social cohesion within communities influence the choice of crime locations [16,17,18,19,20,21,22,23,24]. When infection with COVID-19 leads to isolation, people may not be allowed to enter or leave isolated areas, thus limiting the routine activities of offenders and victims [25]. In addition to isolation, infections may also increase the risk rating of a broader area and lead to the implementation of various lockdown measures, including the closing of stores and business establishments. The closing of facilities would reduce the number of crime generators and crime attractors [26], affecting, in turn, the offenders’ choices of targets [27]. The interplay of the above factors could limit the overall number of crime opportunities, thus affecting crime in general.

Virtually all of the aforementioned studies were conducted in Western cities. Only a few were conducted in a Chinese setting. For example, two studies by Jin et al. demonstrated that Chinese crime statistics in 2020 showed a significant decline in burglary and robbery during the COVID-19 pandemic, but they predicted an increase in 2021 [28,29]. These studies were carried out at the national level. There have been no studies linking crime and COVID-19 to reveal and explain intracity variations. Furthermore, existing studies have focused on the comparison between the lockdown period and the pre- and postlockdown periods. None of them touched on the fine variations during the lockdown. This paper aims to fill these gaps by examining the relationship between violent crime (including assault, provocation, intentional injury, and robbery) and the COVID-19 lockdown policies in ZG City. It compares not only the lockdown period with the nonlockdown periods, but it also compares rates within various stages during the lockdown.

## 2. The Study Area

**Figure 1:**
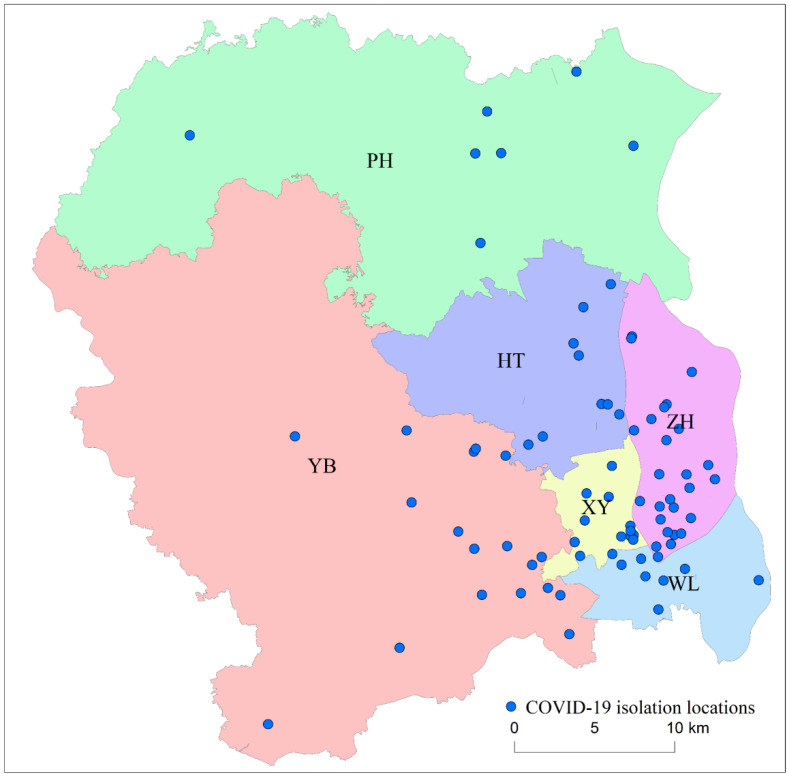
COVID-19 isolation locations, with colored areas representing districts in ZG City, China.

As one of the largest international transportation hubs in China, ZG is a core city of the Pearl River Delta. It has a population exceeding 20 million. Large passenger flows contribute to the transferability of the COVID-19 virus, making it an ideal site for investigating the relationship between crime and COVID-19 lockdowns. The study area consisted of six districts: WL, XY, ZH, HT, PH, and YB, which constitute the main central urban area of ZG City. Compared to Western cities, Chinese cities such as ZG experienced very low infection numbers. During the period from 23 February 2020 to 12 April 2020, a total of 85 isolation locations (Figure 1) were released by the government in the study area. Before or after this period, ZG had no reported additional COVID-19 isolation locations during 2020.

Like other Chinese cities, ZG also adopted an extremely strict control and prevention policy. A single infection case in a district led to the isolation of the residential building of the infected person and the surrounding buildings, and it resulted in elevating the risk rating of the entire district from low to medium. Once it had been labeled a medium-risk area, the district had to close many facilities and limit indoor gatherings. All these measures would limit people’s routine activities. The risk levels for individual districts were updated every week, with 0 indicating low risk and 1 medium risk (Table 1). The 8 weekly time periods are labeled t1 to t8.

The violent crime data in this study cover assault, provocation, intentional injury, and robbery. Domestic violence/abuse is excluded in this study because it is different from public crime. Violent crimes not only have a serious impact on the physical and mental health of individuals, but they also have a serious negative impact on social harmony. They represent a high priority for the police department of ZG City.

## 3. Research Questions, Variable Definitions, and Methods

### 3.1. Research Questions

There are two main research questions in this study: How did violent crime change prior to, during, and after the COVID-19 lockdowns in ZG City? What is the relationship between changes in risk-level policy and changes in crime rates during the various stages of a lockdown? To address the first question, the study compared the spatial patterns of crime among four time periods: (a) the prelockdown period: March–April 2019; (b) the lockdown period: March–April 2020; (c) the early postlockdown period: May–June 2020; and (d) the late postlockdown period: July–August 2020. The first period, March–April of 2019, was selected to match the months of the lockdown period, such that the comparison between the two periods would be meaningful and would not suffer from any seasonal fluctuations in violent crime. For the second question, a panel logistic regression model was used to assess the evolution of the relationship between the change in violent crime and the adjustment of district risk ratings over the various stages of the lockdown.

### 3.2. Variable Definition

**Figure 2:**
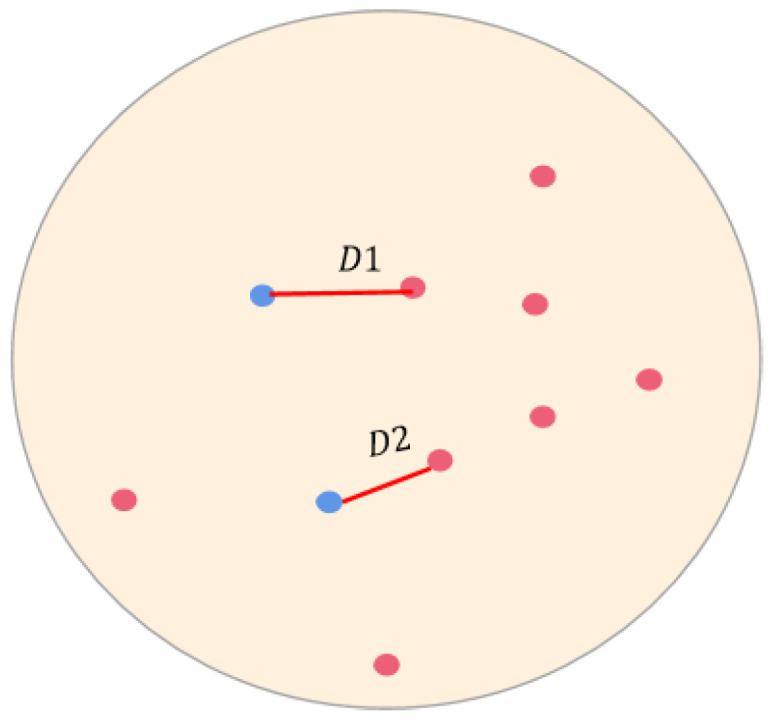
Diagram of the nearest crime distance: blue dots represent isolation locations, red dots are violent crime points, and D indicates the nearest distance to a crime from an isolation location.

For each isolation location, we identified the closest violent crime incidence. The distance, measured in meters, between the two is defined as the nearest crime distance D_i_, where i = [1, 85], representing an isolation location(Figure 2). Each isolation location has a nearest crime distance. For the entire study area, an average nearest crime distance was calculated as $\bar{D}=\frac{D_{1}+\ldots +D_{n}}{n}$, where *n* = 85. A $\bar{D}$ was calculated for each of the four time periods, a, b, c, and d, for Question 1.

For Question 2, we define $D_{\mathrm{it}}$ as the nearest crime distance for isolation location i at time t, where i = [1, 85], and t = [1, 8] (Table 1). Then, a change in the nearest crime distance is defined as ${\Delta D}_{\mathrm{it}}$=$D_{\mathrm{it}}-D_{\mathrm{it}-1}$, i = [1, 85], and t = [2,8]. When $\Delta D_{\mathrm{it}}>0$, that means that crime has moved away from the isolation location; $\Delta D_{\mathrm{it}}<0$ means that crime has moved toward the isolation location. To simplify the research, we were only interested in whether the crime moved away or toward the isolation location. Therefore, all positive $\Delta D_{\mathrm{it}}$ values were changed to 1, and all negative values were changed to 0.

The changes in risk ratings were defined and coded in a similar fashion. We define $R_{\mathrm{it}}$ as the risk rating for isolation location I at time t, where i = [1, 85], and t = [1, 8]. A change in risk ratings is defined as $\Delta R_{\mathrm{it}}\;$ = $R_{\mathrm{it}}\_R_{\mathrm{it}-1}$, where i = [1, 85], and t = [2, 8]. All positive $\Delta R_{\mathrm{it}}$ values were coded as 1, indicating an elevated risk rating; zero and negative $\Delta R_{\mathrm{it}}$ values were coded as 0, indicating an unchanged or downgraded risk rating. Table 2 shows the summary statistics of these variables.

### 3.3. Research Methodology

For Question 1, we mapped the kernel density distributions of violent crime during the four periods. A visual comparison can reveal the overall changes in crime from period a to period d. The nearest crime distances D_i_ are calculated from all of the isolation locations for each time period. The Wilcoxon signed-rank test was used to verify any significant difference among the four sets of D_i_. Furthermore, the averages of the nearest crime distances were compared across the four time periods.

Logit models have also been applied to the study of violent crime [30,31]. The chance ratio OR of the logit model indicates the direction of the relationship between the dependent variable and the explanatory variables. When the OR is greater than 1, the relationship is a positive; when the OR is less than 1, the relationship is negative. A larger deviation of the OR value from 1 suggest a stronger relationship. To answer Question 2, this study used panel logistic regression models to explain changes in the nearest crime distance $\Delta D_{\mathrm{it}}$ by changes in district risk ratings $\Delta R_{\mathrm{it}}$. The panel model uses observations of all time periods for calibration.

## 4. Analysis of Results

### 4.1. Mapping and Testing the Minimal Crime Distance

**Figure 3:**
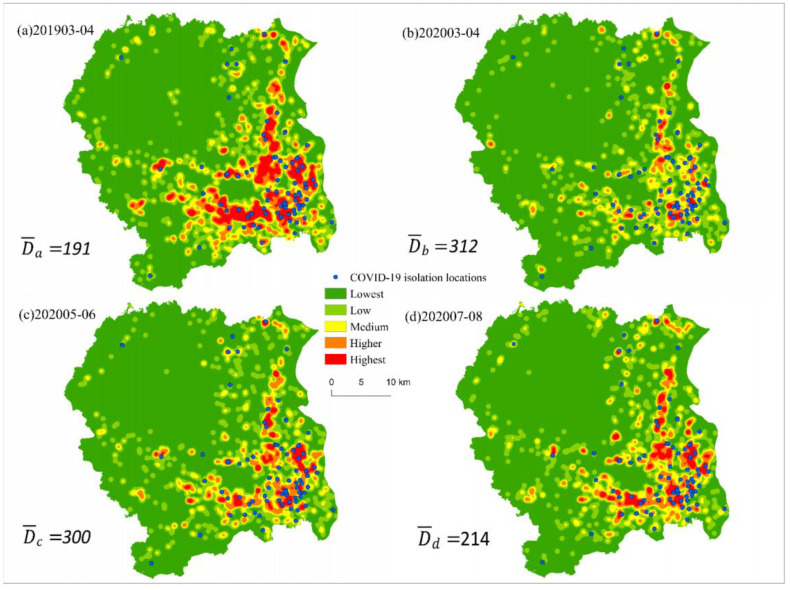
Kernel densities of violent crime during the four time periods: (**a**) pre-COVID-19 pandemic period: March–April 2019; (**b**) Lockdown period: March–April 2020; (**c**) Early post-lockdown period: May–June 2020; (**d**) Late post-lockdown period: June–August 2020. Blue dots indicate isolation locations. The average nearest crime distances are displayed for all four periods.

The kernel density mapping of spatial distributions of violent crime for different periods is shown in Figure 3. The same classifications and legends were applied to all four time periods. It is obvious that the violent crime density was the highest during the pre-lockdown period, lowest during the lockdown period, and the two post-lockdown periods in the middle. This conforms to the U-shaped pattern described in the literature [7,8].

The average nearest crime distances are also displayed on the four panels of Figure 3, showing ${\bar{D}}_{b}>{\bar{D}}_{c}>{\bar{D}}_{d}>{\bar{D}}_{a}$. The average nearest crime distance was the furthest during the lockdown period (Figure 3b) and the closest during the pre-lockdown period (Figure 3a). A comparison of Figure 3a and Figure 3b suggests that the COVID-19 lockdown policy pushed violent crime further away from the isolation locations. A comparison of Figure 3b with Figure 3c and 3d reveals that violent crime slowly but steadily moved back to the isolation locations after the end of the lockdown. The overall change from period a to period d shows a “near—far—near” pattern. This is also in line with the U-shaped curve discussed in previous studies [7,8].

The paper used the Wilcoxon signed-rank test to determine the statistical significance of the differences in the nearest crime distances D_i_ among the four time periods. According to the results of the test shown in Table 3, the pre-lockdown period (a) is significantly different from the lockdown period (b), confirming that the lockdown policy had a significant impact on reducing violent crime. The early post-lockdown period (c) is not significantly different from (b), suggesting the lockdown had a lasting effect after it was lifted. The late post-lockdown period (d) is significantly different from the lockdown period (b), indicating that the lasting lockdown effect stopped, and that violent crime started to return to the pre-lockdown level. These results are consistent with a recent research finding that crime levels rise again after social restrictions are relaxed [7]. The lasting effect after the lift of the lockdown is a new finding that has not been presented by previous studies.

### 4.2. Panel Logistic Model Results: The Impact of Risk Rating Change on the Nearest Crime Change

Results of the panel logistic model on the impact of risk rating change $\Delta R_{\mathrm{it}}\;$on the nearest crime distance change $\Delta D_{\mathrm{it}}\;$are shown in Table 4. According to the results, there is a significant positive relationship between the two. An elevating risk rating pushes violent crime away from the isolation location. Conversely, as the risk level decreases, violent crime moves closer to the isolation location. Such weekly variations during the lockdown have not been reported in the existing literature.

## 5. Conclusions and Discussion

This study tested the relationship between violent crime and COVID-19 lockdown policies in ZG City in southern China. The distance from an isolation location to the nearest violent crime site, called “the nearest crime distance”, is a key variable developed in this study. Kernel density mapping and the Wilcoxon signed-rank test were used to compare the pre-lockdown and post-lockdown periods to the lockdown period. Panel logistic regression was used to test the fine variations among different stages during the lockdown. Several conclusions can be drawn from the study. First, overall violent crime declined during the lockdown, but it bounced back post-lockdown. Second, violent crime moved away from isolation locations during the lockdown. This outward spread continued for the first two months after the lifting of the lockdown, suggesting a lasting effect of the lockdown policy. This finding has not been explicitly reported in the literature. Third, during the lockdown, weekly changes in COVID-19 risk ratings at the district level in ZG City affected changes in the nearest crime distance. In particular, an increase in the risk rating increased that distance, and a drop in the risk rating decreased that distance. Such a finding at a weekly temporal resolution during the lockdown is also a new addition to the literature. In addition to the scholarly contribution, the findings of this could have policy implications for joint crime and pandemic prevention and control.

The “near–far–near” trend of the nearest crime distance from pre-lockdown, to lockdown, to post-lockdown is similar to the findings of Miyar [8] and Payne et al. [12]: that the COVID-19 pandemic had a dampening effect on violent crime. The small rebound in violent crime post-lockdown is also consistent with statistical data and the findings of previous studies [32,33]. However, Boman and Gallupe [15] showed that aggravated assault and homicide either remained the same or increased during the pandemic. The discrepancies between our findings and those of Boman and Gallupe may be related to three reasons. One reason is the difference in crime types. The violent crime data in this study cover assault, intentional injury, and robbery, while homicide is omitted. The second reason may relate to the time difference. This study only covered 4 months post-lockdown. Extending the time period may lead to the identification of a more pronounced bounce-back in violent crime. The third reason may be the difference in policing tactics. The police department of ZG allocated significant resources to combat the COVID-19 pandemic, and the subsequent changes in policing [34,35] may have affected arrest rates [34,35,36]. Exactly how these factors interplay is beyond the scope of this study.

This study found that violent crime moved away from the isolation locations during the lockdown, and this trend lasted for two months after the lockdown was lifted. This lasting effect has not been explicitly reported in the previous literature, as most published studies have focused on an initial reduction in crime under the epidemic, followed by rebound [7,8,12,32,37,38]. The following are two explanations for this lasting effect. First, not all business establishments immediately open right after the end of a lockdown. It takes time for some businesses to return to full operation. Second, people may not fully resume their normal routine activities right after a lockdown ends. The interplay of these two factors affects the overall movement of people, including both potential victims and offenders, which in turn helps to curtail crime.

Weekly changes in COVID-19 risk ratings at the district level in ZG City affected changes in the nearest crime distance from isolation locations during the lockdown. This finding shows that violent crime activities are affected by the volatile COVID-19 epidemic in fine temporal resolutions, such as weeks. When the risk rating for a district increases, the violent crime is reduced near isolation locations in the district during that week. Therefore, the police department can focus its attention on areas further away from the isolation locations. Such focused strategies can be adjusted on a weekly basis.

This study is not without limitations. First, it used the nearest distance to crime from an isolation location as the main measurement for the analysis. Other measurements can be developed to capture a more comprehensive picture [39,40,41,42,43]. Second, this study only explored the effect of risk ratings and lockdowns on crime. Other explanatory variables could be added to the model in future studies. Third, this study only focused on violent crime. Future studies could examine other types of crime as well.

In sum, this study makes an important contribution to the literature on the COVID-19 pandemic. It reveals fine variations during the lockdown. It also presents an intracity study in higher spatial and temporal granularities in the Chinese context. In addition to the scholarly contributions, the findings of this study could have policy implications for joint crime and pandemic prevention and control. In China, local governments rely on police forces and volunteers to implement lockdowns. Police resources, typically stretched thin during lockdowns, are therefore mainly devoted to fighting more violent crimes, which are the focus of this study. It is challenging for the police department to balance the needs of implementing a lockdown and fighting crime. The nuanced relationships in fine temporal resolutions between violent crime and lockdowns revealed in this paper help to dynamically optimize such a balance.

## Figures and Tables

**Table 1:** Risk levels by district in ZG City.

	Time	t1	t2	t3	t4	t5	t6	t7	t8
Region									
XY		0	0	1	1	0	0	1	0
ZH		0	1	1	0	0	0	0	0
WL		0	1	1	0	0	0	0	0
HT		0	1	1	0	0	0	0	0
YB		0	1	1	0	0	0	1	0
PH		0	1	1	0	0	0	0	0

**Table 2:** Change in crime distance and change in risk level.

Variables	Mean	Std. Dev.	Min.	Max.
$D_{\mathrm{it}}$	1096	1160	0	14,745
$\Delta D_{\mathrm{it}}$	0.403	0.491	0	1
$\Delta R_{\mathrm{it}}$	0.171	0.376	0	1

**Table 3:** Results of Wilcoxon signed-rank test.

Distance	a and b	c and b	d and b
Z	−4.127+	−0.603+	−2.710+
P	0.000 ***	0.546	0.007 **

Note: + based on positive order; *** *p* < 0.001, ** *p* < 0.01.

**Table 4:** Results of Wilcoxon signed-rank test.

$\Delta D_{it}>0$	OR	Std. Dev.	*t*-Value	*p*-Value	Significance
$\Delta R_{\mathrm{it}}>0$	1.68	0.344	2.53	0.01	**
Constants	0.62	0.05	−5.59	0	***
AIC	916.50				

Note: ** *p* < 0.01, *** *p* < 0.001.

## Data Availability

Due to an agreement with the crime data provider, we are not allowed to disclose the crime data. The COVID-19 risk levels for individual districts and COVID-19 epidemic data are available to the general public on the website of the local health commission.
